# Duodenal Enteroendocrine I-Cells Contain mRNA Transcripts Encoding Key Endocannabinoid and Fatty Acid Receptors

**DOI:** 10.1371/journal.pone.0042373

**Published:** 2012-08-02

**Authors:** Alexandros G. Sykaras, Claire Demenis, R. Maynard Case, John T. McLaughlin, Craig P. Smith

**Affiliations:** 1 Faculty of Life Sciences, The University of Manchester, Manchester, United Kingdom; 2 Graduate Programme “Molecular Basis of Human Diseases”, Faculty of Medicine, University of Crete, Heraklion, Crete, Greece; 3 School of Translational Medicine, School of Medicine, The University of Manchester, Manchester, United Kingdom; University of Ulster, United Kingdom

## Abstract

Enteroendocrine cells have a critical role in regulation of appetite and energy balance. I-cells are a subtype of enteroendocrine cells localized in duodenum that release cholecystokinin in response to ingested fat and amino-acids. Despite their potentially pivotal role in nutrient sensing and feeding behaviour, native I-cells have previously been difficult to isolate and study. Here we describe a robust protocol for the isolation and characterization of native duodenal I-cells and additionally, using semi-quantitative RT-PCR we determined that mouse duodenal I-cells contain mRNA transcripts encoding key fatty acid and endocannabinoid receptors including the long chain fatty acid receptors GPR40/FFAR1, GPR120/O3FAR1; short chain fatty acid receptors GPR41/FFAR3 and GPR43/FFAR2; the oleoylethanolamide receptor GPR119 and the classic endocannabinoid receptor CB1. These data suggest that I-cells sense a wide range of gut lumen nutrients and also have the capacity to respond to signals of fatty-acid derivatives or endocannabinoid peptides.

## Introduction

Endocrine cells distributed throughout the intestinal tract integrate dietary and pathological cues and, via hormonal and neural signals, orchestrate multiple tissues to co-ordinate food digestion and regulate appetite. Collectively these cells are termed enteroendocrine (EEC) cells and they constitute ∼1% of the intestinal epithelial cell population [1], [2], [3], [4], [5].

I-cells are a subset of duodenal EEC cells that express the anti-orexigenic and principal satiety peptide hormone cholecystokinin (CCK) [6], [7], [8]. CCK is released by I-cells in response to luminal nutrients, in particular fatty acids and amino acids [9]. CCK co-ordinates digestion by inhibiting gastric emptying, and by stimulating gallbladder contraction and pancreatic enzyme secretion [10]. I-cells are therefore pivotal in the intestinal response to nutrients in so far as they are suggested to sense luminal gut nutrients by membrane bound G-protein coupled receptors (GPCRs) [2], [11], integrate nutrient signals and transmit these signals both centrally and peripherally by hormone release, and to the brain by vagal afferent-mediated signalling.

Transcripts encoding the long chain fatty acid receptors (LCFA) free fatty acid receptor 1 (FFAR1, formerly known as GPR40) [12], [13] and omega-3 fatty acid receptor 1 (O3FAR1, formerly known as GPR120) [14], [15] are present in I-cells [11]. Signalling by GPR40/FFAR1 has been suggested to regulate CCK release from I-cells [11]. Interestingly, in humans we have reported release of CCK in response to intragastric fatty acids with chain lengths matching the ligand profiles of GPR40/FFAR1 and GPR120/O3FAR1 [16]. In addition to GPR40/FFAR1 and GPR120/O3FAR1, other GPCRs have been implicated in EEC cell nutrient sensing and appetite regulation. These include the short chain fatty acid (SCFA) receptors free fatty acid receptor 3 (FFAR3, formerly known as GPR41) and free fatty acid receptor 2 (FFAR2, formerly known as GPR43) [17], [18], [19]. GPR41/FFAR3 is highly enriched in duodenal and colonic L-cells [19] and also in CCK-containing cells of the small intestine [20]. It has been proposed that GRP41/FFAR3 acts as a sensor of SCFA generated by bacterial fermentation of polysaccharides [20]. GPR43/FFAR2 is expressed in duodenal and colonic L-cells and mediates GLP-1 release in response to SCFA [19].

GPCRs belonging to the endocannabinoid receptors family are also known to be expressed in the small intestine, but their cellular distribution within duodenal epithelium remains undetermined. These include GPR119 that binds oleoylethanolamide (OEA), an anorectic lipid amide that is a derivative of fat digestion [21] and 2-oleoyl glycerol, a product of digestion of dietary triacylglycerol [22]. Activation of GPR119 stimulates glucagon-like peptide 1 (GLP-1) release from L-cells [21], [23], [24], enhances glucose-stimulated insulin secretion and inhibits gastric emptying [25], [26], [27]. In addition to GPR119, the cannabinoid receptor 1 (CB1) is a GPCR that has a key role in the regulation of appetite. There is evidence that CB1 is expressed in vagal afferent neurones where it mediates the transmission of orexigenic signals to brain [28], [29], [30], but its expression in duodenal epithelium remains obscure.

The study of enteroendocrine cells is difficult because of their diffuse and sparse distribution, and their relatively indistinct morphology. In the past, research has focused on surrogate models, such as the enteroendocrine cell lines STC-1 and GLUTag, that are at best approximations of native enteroendocrine cells. The recent engineering of transgenic mouse models with genetically tagged genes that encode gut hormones, enabling fluorescent delineation of native EEC cells, has ushered in a new era of EEC research [11], [31], [32], [33], [34], [35], [36]. In this study we describe a robust method to isolate and purify I-cells and use these purified populations to probe the I-cell transcriptome for key nutrient sensors and endocannabinoid receptors. As a proof of principle, we perform a targeted gene expression analysis of I-cells. Our results confirm that duodenal I-cells contain mRNA transcripts that encode LCFA receptors GPR40/FFAR1 and GPR120/O3FAR1 and are highly enriched in mRNA transcripts of SCFA receptors (Gpr41/Ffar3 and Gpr43/Ffar2) and the endocannabinoid receptors Gpr119 and CB1.

## Results

### Validation of Experimental Model/Imaging of Duodenal I-cells

**Figure 1:**
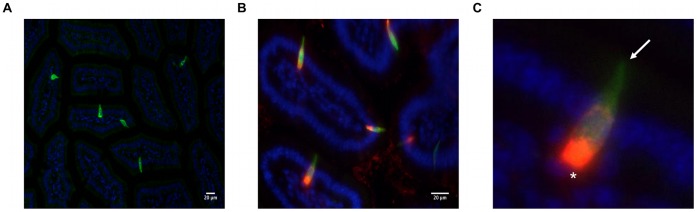
eGFP expressing cells are strongly immunopositive for CCK. Representive images of transverse sections of duodenum from CCK-eGFP mice. A: eGFP-tagged cells (green) were flask-shaped with a diffuse distribution, characteristic of ‘open-type’ enteroendocrine cell. B: Immunostaining with anti-CCK antibody (red) showed colocalization of CCK and eGFP in ∼ 90% of eGFP-labelled cells. C: High magnification micrograph (2.5X digital zoom) revealed I-cells had a narrow apical membrane (arrow) that was in contact with gut lumen and a broad basolateral membrane (asterisk) with adjacent strong CCK staining. In all images, nuclei were counter-stained with Hoechst 33342 (blue).

In order to isolate duodenal I-cells, we used a BAC (Bacterial Artificial Chromosome) transgenic mouse strain which expresses an eGFP reporter regulated by CCK promoter elements. We first examined cryosections of duodenum and observed that eGFP expression was restricted to cells representing less than 1% of epithelial cell population (counting slides from 3 mice) that lay intermittently between intestinal epithelial cells (enterocytes). eGFP-labeled cells had a flask-shaped morphology typical of enteroendocrine cells; characterized by a narrow apical membrane in contact with the lumen and a much broader basolateral membrane (Figure 1A). The contact of the apical membrane with the lumen meant that the eGFP expressing cells were “open-type” cells. To validate that eGFP specifically labels CCK-expressing cells, we performed immunostaining with a proven CCK-specific antibody and showed that eGFP expression colocalized with CCK staining (Figure 1B). Counting of eGFP-positive and CCK-positive cells in multiple duodenal slides revealed that 88.4% (SEM ±1.25, n = 19 slides-prepared from 3 mice) of eGFP-tagged cells expressed CCK. This finding confirmed that the CCK promoter transgene faithfully marked CCK-containing I-cells. The CCK-staining pattern we observed was characteristic of I-cells in that it was very intense towards the basal pole of the cell where the CCK containing secretory vesicles congregate in preparation for release (Figures 1B, 1C).

### FACS Sorting of eGFP+ Cells (I-cells)

**Figure 2:**
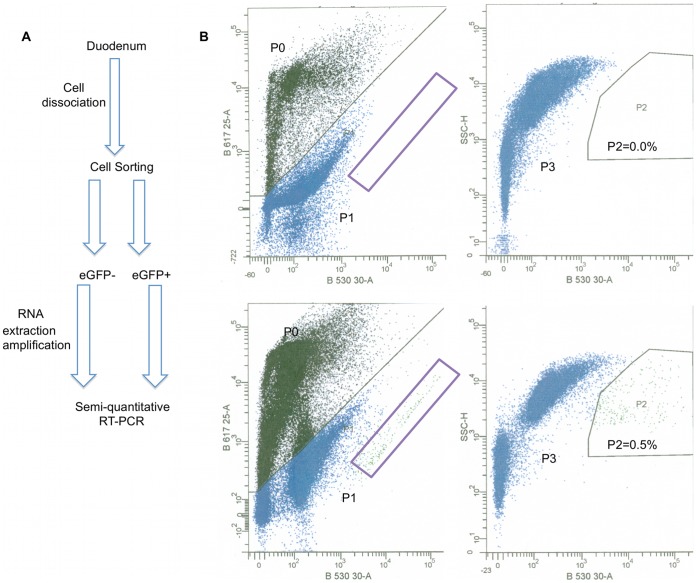
FACS-sorting of eGFP+ and eGFP− cells. A: Schematic overview of the methods employed to isolate I-cells and perform transcript analysis. B: Dissociated duodenal cells from eGFP-CCK mice were sorted using a BD FACS Aria cell sorter (lower panels). Dissociated duodenal cells from a WT/CD-1 mouse were used as a control to set gating parameters for analysis and sorting (upper panels). Cells were first stained with PI and analyzed by eGFP (X-axis) and PI (Y-axis) fluorescence intensity (left panels). PI- positive cells (dead cells, P0) were excluded from further analysis. Live cells (Blue, P1) from CCK-eGFP mice displayed a population of highly fluorescent cells (purple rectangle) that were not present in cell from WT mice. Live cells were also analyzed based on eGFP fluorescence intensity (X-axis) and side scatter (Y-axis). Cells that expressed eGFP were gated as eGFP+ cells, representing 0.5% of total cell population (P2, right lower panel). This population was not present in cells from WT mice (P2, right upper panel). All other cells (P3) were gated as eGFP−. eGFP+ cells and approximately equal number of eGFP− cells were sorted.

We used a chemical method (Protocol A/Protocol B described in Methods section) to dissociate duodenal epithelium into a single-cell population that was then subjected to FACS analysis in order to isolate eGFP+ cells (Figure 2A). Both protocols yielded very similar results in terms of the: a) morphology of dissociated cells; b) viability of dissociated cells (there was a high variability in the viability of dissociated cells in each method but not any clear difference in the cell viability between the two methods); c) forward and side scatter profile of cell population analyzed by FACS; d) percentage of eGFP+ cells; e) number of sorted eGFP+ f) quality of RNA prepared from sorted cells. Imaging of dissociated cells revealed that there was no discernable difference in morphology between isolated eGFP+ enteroendocrine and epithelial cells. In each optical field, we observed eGFP+ cells that displayed different levels of fluorescence intensity (Figure S1A). These differences were evident even in preparations of dispersed cells from a single mouse. Dissociated cells from wild-type and CCK-eGFP mice were exposed to propidium iodide (PI) prior to FACS analysis, to eliminate dead cells. Gating of live cells (P1) revealed a sub-population of highly fluorescent cells (P2) that were present only in cells prepared from transgenic mice (Figure 2B). Sorted eGFP+ cells represented 0.3–0.7% of the viable epithelial cell population in the majority of FACS sorting experiments we performed. The number of eGFP+ cells sorted from four mice was typically between 5000–15000 and this depended on the initial viability of dissociated cells prior to FACS.

We used epi-fluorescence microscopy to check the purity of FACS sorted eGFP+ cells. By visualizing sorted cells, we estimated the purity of sorted eGFP+ cells routinely to be above 90% (Figure S1A, B, C).

### Sorted eGFP+ Cells Represent I-cells

**Figure 3:**
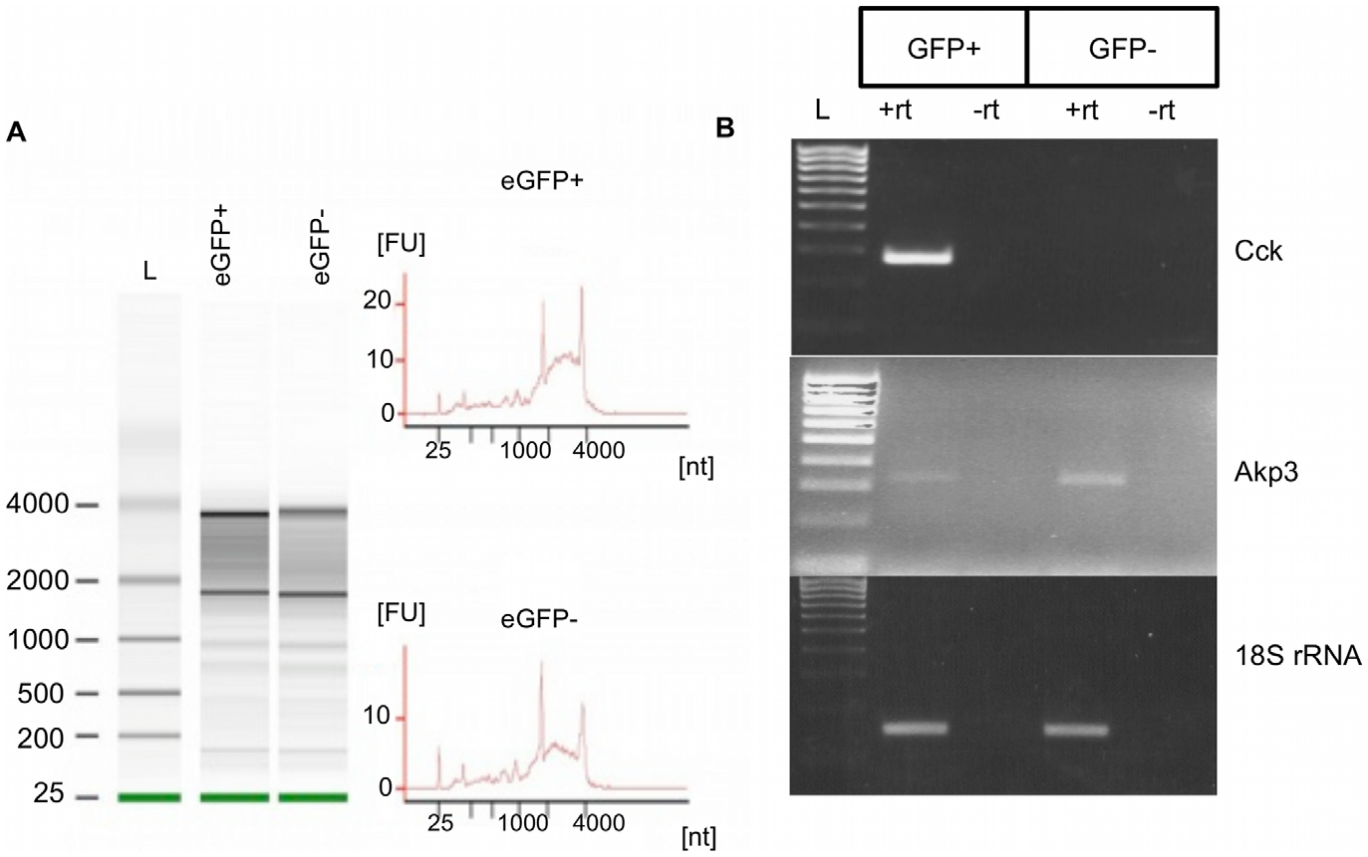
eGFP+ sorted cells represent a highly enriched population of I-cells. A: Analysis of RNA extracted from eGFP+ and eGFP− cells. RNA was analyzed using a RNA 6000 PicoChip kit. Intact 28S and 18S ribosomal bands (gels, left panel) or distinct 28S and 18S RNA ribosomal peaks (electropherograms, right panel) confirmed integrity of extracted RNA from sorted cells. L indicates Pico 6000 RNA ladder that spans 0.2–6.0 kb. B: eGFP+ cells were highly enriched in Cck mRNA transcripts. Semi-quantitative PCR for Cck mRNA demonstrated that only eGFP+ cells contain Cck mRNA transcript, a marker of I-cells. Akp3 mRNA transcript, a marker of enterocytes, was mainly detected on eGFP− cells, showing little contamination of eGFP+ cells (I-cells) with enterocytes. 18S rRNA was used as a loading control. L declares Hyperladder IV (100–1000 bp, Bioline, UK).

We used RT-PCR for Cck mRNA, a transcript that is the defining characteristic of I-cells, to confirm that enriched eGFP+ cells truly represent I-cells. RNA was prepared from eGFP+ and eGFP− cells. Since RNA extraction from FACS-sorted cells may result in partially or totally degraded RNA, and this degradation may compromise the results of subsequent applications such as RT-PCR, we verified the integrity of RNA prepared from eGFP+ and eGFP− using a RNA 6000 PicoChip on an Agilent 2100 Bioanalyzer. The presence of intact ribosomal bands (28S and 18S RNA) in each sample (RIN score >5) confirmed the integrity of purified RNA (Figure 3A). Using RT-PCR, *Cck* transcripts were only detected in eGFP+ cells and thus confirmed that eGFP+ cells represent very highly enriched I-cells (Figure 3B). RT-PCR for the enterocyte marker gene *Akp3* (intestinal alkaline phosphatase 3) [37] showed that this transcript was highly expressed in eGFP− cells, with a much lower expression level in eGFP+ cells, indicating a minor component of enterocytes in the eGFP+ fraction (Figure 3B).

To confirm that sorted eGFP− cells were depleted of I-cells, we compared the amount of Cck mRNA transcripts in eGFP− sorted cells with unsorted dissociated duodenal cells. We detected Cck mRNA in unsorted dissociated duodenal cells, but not in eGFP− cells (Figure S2A), indicating that eGFP− cells were depleted of I-cells. Enrichment of I-cells in eGFP+ sorted cells was confirmed by comparing the amount of Cck mRNA transcript in eGFP+ sorted cells with unsorted dissociated duodenal cells (Figure S2B).

### I-cells Contain mRNA Transcripts Encoding LCFA Receptors (GPR40/FFAR1, GPR120/O3FAR1), SCFA Receptors (GPR41/FFAR3 and GPR43/FFAR2) and Endocannabinoid Receptors (GPR119 and CB1)

**Figure 4:**
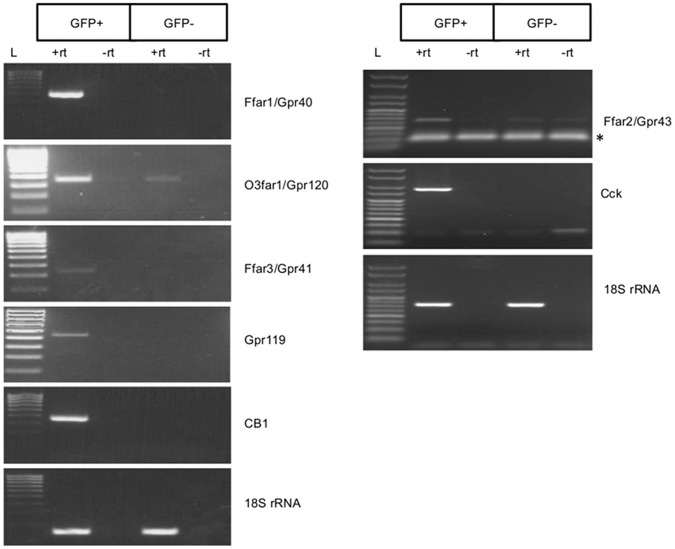
I-cells contain GPR40, GPR120, GPR41, GPR43, GPR119 and CB1 mRNA transcripts. Semi-quantitative RT-PCR analysis revealed that eGFP+ cells (I-cells) were enriched in mRNA transcripts encoding Gpr40/Ffar1, Gpr41/Ffar3, Gpr43/FFAR2, Gpr119 and CB1 whereas Gpr120/O3far1 was enriched in eGFP+ cells, but was also present eGFP− cells; RT-PCR of 18S rRNA confirms that equal amount of cDNA template from eGFP+ and eGFP− cells was used for the analysis. L declares Hyperladder IV (100–1000 bp, Bioline, UK). Asterisk indicates bands representing primer dimers.

We used semi-quantitative RT-PCR to determine the presence in I-cells of mRNA transcripts encoding key GPCRs involved in nutrient sensing (Figure 4). Transcripts encoding LCFA receptors GPR40/FFAR1 and GPR120/O3FAR1 were expressed in I-cells: Gpr40/Ffar1 mRNA was highly enriched in I-cells and could not be detected in non I-cells (eGFP− cells); Gpr120/O3far1 mRNA was detected in both I-cells and non I-cells, though the amount of amplified product showed that this target was enriched in I-cells and therefore unlikely to be due to any contaminating cDNAs from non I-cells; SCFA receptor Gpr41/Ffar3 mRNA transcript was found to be abundant in I-cells and not present in non I-cells.

We then determined if OEA receptor Gpr119 and endocannabinoid receptor CB1 mRNA transcripts were present in I-cells. First, we detected transcripts for both Gpr119 and CB1 in the mixed populations of dissociated cells so then we proceeded to analyze their expression pattern in I-cells. RT-PCR showed that Gpr119 and CB1 mRNA transcripts were specifically expressed in I-cells and were not detectable in non I-cells (Figure 4, left panel). In an independent sorting experiment, we performed semi-quantitative RT-PCR for Gpr43/Ffar2 and found that this transcript was present in I-cells (Figure 4, right panel).

### Confirmation of GPCRs Enrichment by Semi-quantitative RT-PCR on Amplified cDNA from eGFP+ and eGFP− cells

**Figure 5:**
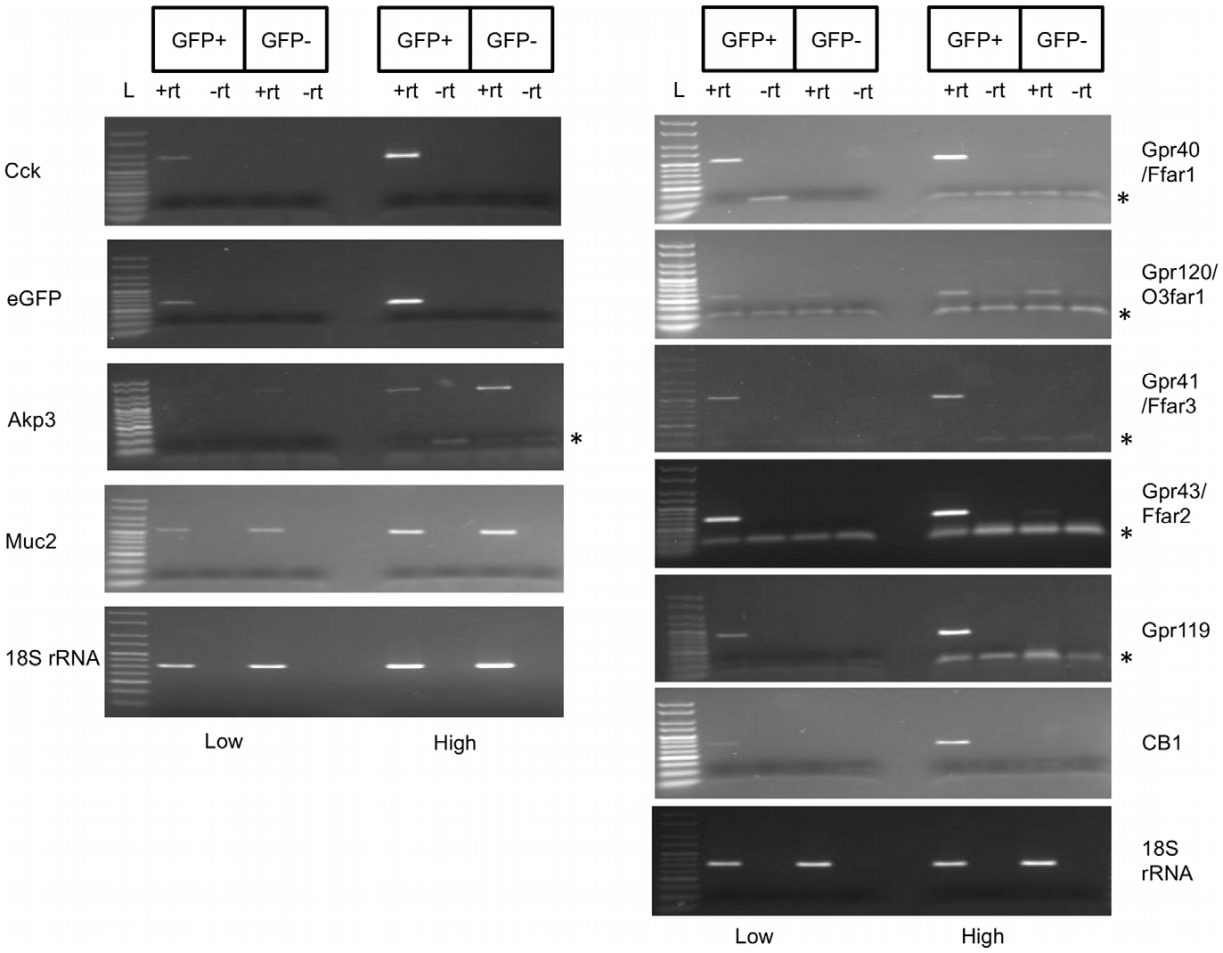
Semi-quantitative RT-PCR on amplified cDNA from sorted cells validates I-cells enriched mRNA transcripts. Semi-quantitative RT-PCR analysis using amplified cDNA from eGFP+ and eGFP− cells. Left panel: eGFP+ cells were highly enriched in mRNA transcripts encoding eGFP and CCK whereas these transcripts were not detected in eGFP− cells. eGFP− cells were enriched in mRNA transcripts encoding AKP3 and MUC2, markers of enterocytes and goblet cells respectively. Right panel: Validation of Gpr40/Ffar1, Gpr120/O3far1, Gpr41/Ffar3, Gpr43/Ffar2, Gpr119 and CB1 mRNA transcripts enrichment in I-cells. 18S rRNA was used as a loading control. PCR products were amplified simultaneously, using different number of cycles (low-for lower number of cycles and high-for higher number of cycles). Equal volumes of each reaction were run on the same 2% agarose gel. L declares Hyperladder V (50–250 bp, Bioline, UK). Asterisk indicates primer dimers.

Because the amounts of RNA isolated from FACS sorted cells were limiting, we engineered an amplified cDNA library from sorted eGFP+ and eGFP− RNA to enable validation of our RT-PCR analysis and for future transcriptome analysis. RNA/cDNA amplification generated more than 5 µg of cDNA from a starting material of 2–5 ng total RNA. We then performed detailed independent semi-quantitative PCR analysis (with different sets of primers) on amplified cDNA. Our analysis of eGFP+ and eGFP− cDNA verified that eGFP+ cells exclusively contained both Cck and eGFP mRNA transcripts compared to eGFP− cDNA pools confirming enrichment of I-cells and suggesting that eGFP− cells were not contaminated with I-cells. Thus, the exclusive presence of a transcript in eGFP+ cells strongly suggests that the encoded protein is expressed in I-cells. We also performed PCR for transcripts that are characteristic of enterocytes (*Akp3*) and goblet cells (*Muc2*, mucin 2) [38], and showed that they are much more abundant in eGFP− cells than eGFP+ cells. This result demonstrated that eGFP+ do not represent a 100% pure population of I-cells but a highly-enriched I-cells population with little contamination from epithelial and goblet cells (Figure 5, left panel).

Semi-quantitative RT-PCR results confirmed our previous findings. Gpr40/Ffar1 is highly enriched in eGFP+ cells and not present in non I-cells, whereas Gpr120/O3far1 is present in both cell populations but enriched in I-cells. I-cells are also highly enriched in mRNA transcripts of SCFA receptors Gpr41/Ffar3 and Gpr43/Ffar2. Using the amplified cDNA pools, we also confirmed that endocannabinoid receptors Gpr119 and CB1 mRNA transcripts are highly enriched in I-cells (Figure 5, right panel).

## Discussion

In this study, we developed a rigorous and reproducible method for the isolation of duodenal I-cells and performed a targeted transcriptomic analysis to determine if I-cells expressed GPCRs that have been ascribed pivotal roles in sensing of fatty acids, endocannabinoid peptides or lipid amide derivatives.

First, we validated the fidelity of the transgene expression in the CCK-eGFP mouse (Tg(CCK-EGFP)BJ203Gsat/Mmmh) duodenum. eGFP-tagged cells were observed to be diffuse, rare (less than 1%) and have the morphological characteristics of classic ‘open type’ EEC cells. We noted that eGFP+ cells displayed a spectrum of different levels of eGFP fluorescence, probably due to differential activity of the CCK promoter. Immunohistochemistry verified that the majority (approximately 90%) of eGFP-tagged cells expressed CCK, thus confirming the reliability of this animal model for the study of I-cells. It is possible that the expression level of CCK in the ∼10% of cells that expressed eGFP, but that were not stained by the CCK antisera, was very low and below the detection limit of the immunostaining method we employed. In agreement with other studies [11], [33], [35], FACS analysis showed that eGFP+ cells represented 0.3–0.7% of the total duodenal cell population.

Using FACS, we sorted populations of eGFP+ and eGFP− cells and successfully extracted RNA of high quality from these cells. RT-PCR for CCK mRNA, the characterizing marker of I-cells, and eGFP, the resident transgene, confirmed that sorted eGFP+ cells represented a highly enriched population of I-cells. RT-PCR for marker genes of possible contaminating enterocytes or goblet cells revealed that the eGFP+ cell population although not devoid of contamination, represented a highly enriched I-cell population with little noise from enterocytes and goblet cells. These results instilled confidence that subsequent amplification of PCR products from eGFP+ cDNA pools were not a result of contamination from enterocytes or goblet cells but representative of the I-cells transcriptome.

mRNA transcripts encoding the LCFA receptors Gpr40/Ffar1 and Gpr120/O3far1 have been reported to be present in EEC cells and specifically I-cells [11], [39]. Our results showing the enrichment of Gpr40/Ffar1 and Gpr120/O3far1 mRNA transcripts in I-cells are confirmatory of our successful purification of I cells using the method we developed. Presence of Gpr40/Ffar1 and Gpr120/O3far1 mRNA transcripts in I-cells support the concept that activation of these nutrient sensors triggers or modulates CCK release in response to LCFA [11], [40]. GPR40/FFAR1 has been suggested to be responsible for mediates fatty-acid induced CCK secretion in native I-cells [11]. In addition, GPR120/O3FAR1 has been reported to be responsible for triggering fatty-acid induced CCK secretion in the I-cell surrogate model STC-1 cells [40]. Our finding that Gpr120/O3far1 mRNA is present in non I-cells may indicate a function for this receptor in enterocytes where it may function to co-ordination of lipid absorption. Gpr120/O3far1 mRNA transcript is also present in macrophages [15], so its presence in intestinal macrophages may also explain its detection in non I-cells cDNA preparation. Clearly, functional studies are needed on native I-cells to clarify the contribution of Gpr40/Ffar1 and Gpr120/O3far1 in modulating fatty-acid induced CCK secretion by I-cells.

In the present study we report for the first time that duodenal I-cells are highly enriched in mRNAs encoding the SCFA receptors GPR41/FFAR3 and GPR43/FFAR2. Previously, Samuel et al [20] showed that Gpr41/Ffar3 mRNA was expressed in small intestine with highest expression in ileum and reported that Gpr41/Ffar3 was highly enriched in CCK-containing enteroendocrine cells isolated from small intestine. Our results refine this latter observation by showing that Gpr41/Ffar3 mRNA is present in duodenal I-cells. Additionally, we show that Gpr43/Ffar2 mRNA is highly enriched in I-cells. Without definitive knowledge of the localization of GPR41/FFAR3 and GPR43/FFAR2 within the I-cells, in particular whether the receptors sense luminal ligands or basolateral (plasma) ligands, it is difficult to ascribe a possible function to GPR41/FFAR3 and GPR43/FFAR2. However, it has been shown that CCK is released only in response to LCFA, but not SCFA [16], [41], so it is unlikely that GPR41/FFAR3 or GPR43/FFAR2 in I-cells have a critical role as nutrient sensors regulating CCK secretion. On the other hand, there is evidence that GPR41/FFAR3 mediates secretion of enteroendocrine hormone PYY in response to SCFA generated from fermentation of dietary fibre by gut microbiota [20]. Thus, GPR41/FFAR3 regulates intestinal transit time (PYY delays the digestion process), absorption of SCFA and hepatic lipogenesis and is suggested to play a critical role as a link that connects gut microflora and energy balance. GPR41/FFAR3 and GPR43/FFAR2 also mediate GLP-1 release upon stimulation by SCFA, regulating glucose tolerance. Interestingly, GLP-1 release seems to be dependent on a Gq-mediated signalling pathway indicating a potentially crucial role of GPR43/FFAR2 [19]. Additionally, a study by *Lin et al* reported that GPR41/FFAR3 is not involved in butyrate or propionate-induced GIP release and only partially mediates GLP-1 release in response to butyrate [42]. These findings may suggest that GPR43/FFAR2 has an important role in SCFA-induced release of gut hormones. It is intriguing that duodenal I-cells, like small intestinal L-cells [19], are highly enriched in Gpr41/Ffar3 and Gpr43/Ffar3 mRNA transcripts, although the concentration of SCFA in the duodenal micro-environment is very low in comparison with distal ileum/colon where, due to bacterial metabolism SCFA are present in high concentrations. With these findings in mind, GRP41/FFAR3 and GPR43/FFAR2 in duodenal I-cells may not function as apical projecting gut lumen sensors, but sense circulating SCFA in plasma to modulate I-cell function. The availability of specific antisera targeting GPR41/FFAR3 and GPR43/FFAR2 may answer this intriguing question.

Another GPCR mRNA transcript enriched in duodenal I-cells is Gpr119. Gpr119 mRNA expression has been previously reported in L- [23], [32] and K-cells [31]. GPR119 can be classified as an endocannabinoid receptor that is activated by fatty-acid derived ethanolamides, such as the anandamide-related peptide OEA, and mediates GLP-1 release from L-cells [23], [24]. It is an open question whether binding of OEA or other natural acylethanolamide to GPR119 expressed in I-cells results in CCK secretion. OEA has been reported as a satiety messenger that is mobilized from intestinal epithelium in response to fat ingestion [43]. This satiety pathway is CD36-dependent and CCK-independent. The presence of Gpr119 in I-cells may indicate a link between the two satiety pathways (CCK-dependent and CCK-independent) that are activated after fat ingestion.

The presence of Gpr119 mRNA in I-cells led us to demonstrate that the classic endocannabinoid receptor CB1 mRNA transcript is highly expressed in I-cells. CB1 is expressed in vagal afferents [30], but its expression in intestinal epithelial cells is controversial [44], [45]. We found that I-cells are highly enriched in the CB1 mRNA transcript. Our finding may suggest that intestinal endocannabinoids regulate I-cell function. DiPatrizio *et. al* recently reported that intestinal endocannabinoids were elevated in response to a fatty meal [46]. This response, which was dependent on the orosensory properties of the fatty meal, was mediated by vagal afferents and was found to stimulate fat intake by signalling via CB1 receptors [46]. In a recent commentary Di Marzo suggested that endocannabinoids might inhibit CCK release from duodenal enteroendocrine cells, and thereby stimulate food intake [47]. Our finding that CB1 mRNA is present in I-cells supports this hypothesis and may provide a means by which the local endocannabinoid system can regulate satiety pathways possibly by modulating CCK release.

By comparing the results of the current study with that of others detailing expression profiles of duodenal/proximal small intestine EEC cells, it can be seen that the expression profile of nutrient sensors in different types of EEC cells is very similar. Studies from Gribble, Reimann and colleagues have shown proximal intestinal L-cells to be highly enriched for mRNA transcripts encoding Gpr40/Ffar1, Gpr41/Ffar3, Gpr119 and Gpr120/O3far1 [32]. Duodenal K-cells are also highly enriched in mRNA transcripts encoding Gpr40/Ffar1, Gpr119 and Gpr120/O3far1 [31]. This common repertoire of nutrient sensor GPCRs may suggest that enteroendocrine cells localized in the same segment of the intestine share similar mechanisms of nutrient sensing. A recent paper from the Gribble/Reimann group reported an extensive overlap between the hormone content of upper small intestinal L- and K- indicating that these EEC subtypes may have a similar profile of receptors that recognize nutrients and regulate hormone secretion [48].

In summary, we have developed a protocol for the isolation of duodenal I-cells. We have employed this approach to sort I-cells and perform targeted transcriptomic analysis of mRNA transcripts that encode nutrient sensors and endocannabinoid receptors. RT-PCR analysis was performed directly on unamplified or amplified cDNA from FACS sorted cells, with similar results. Our data suggest that mRNA transcripts encoding the LCFA receptors Gpr40/Ffar1 and Gpr120/O3far1, SCFA receptors Gpr41/Ffar3 and Gpr43/Ffar2, lipid amides receptor Gpr119 and endocannabinoid receptor CB1 are enriched in duodenal I-cells. The presence of these GPCRs transcripts supports the suggestion that they act as nutrient sensors regulating CCK release. The presence of endocannabinoid receptors mRNA transcripts in I-cells may indicate that the endocannabinoid system modulates nutrient sensing and influences CCK release. Functional studies are required to determine how activation of these nutrient sensors mediate CCK release and to understand the role of the intestinal endocannabinoid system in the regulation of nutrient sensing and CCK secretion from duodenal I-cells.

## Methods

### Ethics Statement

All animal procedures used in this study were ethically approved by the University of Manchester Ethical Review Process Committee, in accordance with the UK Home Office regulations, under licence 40/3409. All animal procedures used in this study were in accordance with Animals Scientific Procedures Act 1986 (UK) and UK Home Office regulations.

### Experimental Animals

CCK-eGFP mice were purchased from MMRRC (Mutant Mouse Regional Resource Center, USA). CCK-eGFP (Tg(CCK-EGFP)BJ203Gsat/Mmmh) is a transgenic mouse model that expresses enhanced Green Fluorescence protein (eGFP) under the control of *Cck* gene promoter and was generated as part of the GENSAT (Gene Expression Nervous System Atlas) project at Rockefeller University [49]. Mice were bred in-house and were kept on a 12 h light: dark cycle with ad libitum food and water. Adult mice (8 to 16 weeks old) were used for all the experiments. The presence of the transgene was verified by genotyping, according to the instructions provided by MMRRC (http://www.mmrrc.org/catalog/sds.php?mmrrc_id=249).

### Imaging of CCK-eGFP Cells

Cryosections were prepared from mouse duodenum, fixed in 4% paraformaldehyde (PFA) and cryoprotected overnight in 30% sucrose/PBS. 6 µm cryosections were mounted onto superfrost plus slides. Sections were thawed at 60°C for 30 mins, soaked in 4% PFA 15 mins and washed in dH_2_0. Antigen sites were blocked for 30 minutes in 50 mM NH_4_Cl-PBS, and permeabilised in buffer (1% BSA, 0.2% gelatine, 0.05% saponin-PBS). Sections were then incubated overnight with anti-rabbit anti-CCK antiserum L421 at 1/500 dilution in antibody buffer (0.1% BSA, 0.3% Triton-X in PBS). After incubation with primary antibody, sections were returned to room temperature for 60 minutes, sections were washed three times in 0.1% BSA, 0.2% gelatine, 0.05% saponin in PBS buffer, after which goat anti-rabbit IgG Alexafluor594 (A-11012) secondary antibody was applied at 1/1000 dilution in antibody buffer for 90 minutes at room temperature. Slides were washed 3 times in PBS, incubated for 15 minutes with nuclei acid stain Hoechst 33342 (Invitrogen, UK) at a final concentration of 0.5 ng/µl, washed with dH_2_0 and mounted with DAKO glycergel. They were visualised on Olympus BX51 upright microscope using a 20X or 40X objective. Specific band pass (BP) filter sets for Hoechst 33342 (excitation BP 350/50 nm, emission BP 460/50 nm), GFP (excitation BP 480/40 nm, emission 535/50 nm) and Texas Red (excitation BP 560/55, emission 645/75 nm) were used to avoid channel bleed-through. Images were captured using a coolsnap ES camera (Photometrix) through MetaVue Software (Molecular Devices) and processed using ImageJ software (NIH, http://rsbweb.nih.gov/ij/).

### Preparation of Isolated Duodenal Epithelial Cells

Four adult male CCK-GFP mice were used in each experiment. CD-1 adult mice or mice that did not express the transgene (eGFP-negative) were used as control. Mice were anaesthetized with CO_2_ and were killed by dislocation of the neck. Duodenum (first 5–6 cm after pyloric sphincter) was removed and put into ice-cold PBS. Luminal contents were manually removed, the tissue was rinsed with PBS several times and was dissected longitudinally and then cut laterally into 5–10 mm pieces, so as to expose the epithelium. We used a chemical/mechanical method for dissociation of the epithelium. Tissue fragments were placed into 100 mm Petri-dish containing ice-cold calcium-magnesium free Hanks Balanced Salt Solution (CMF-HBSS) supplemented with 5% Fetal Bovine Serum (FBS) and 0.5 mM ditheiothreitol (DTT) and shaken gently for 10 minutes at 4°C. Then, duodenal pieces were transferred into conical tubes containing CMF-HBSS with 5% FBS, 0.6 mM DTT and 1 mM 2,2′, 2″, 2″’-(Ethane-1, 2-diyldinitrilo) tetraacetic acid (EDTA). Epithelium was dissociated into single-cells by shaking at 37°C for 20 minutes (175 rpm for the first 10 minutes and 100 rpm for another 10 minutes). Dissociated single cells were pelleted by centrifugation for 5 minutes at 150–250 g at room temperature. Cell pellet was resuspended into PBS containing 3% FBS and filtered through a 40 µm cell strainer to ensure that only dissociated single cells will be subject to Fluorescence Activated Cell Sorting (FACS) analysis (Protocol A).

An alternative protocol that we have used successfully for the isolation of single-cells is based on the same principle but omits the use of DTT. Instead, tissue fragments were placed directly into conical tubes containing CMF-HBSS supplied with 5% FBS and 1mM and dissociated for 30 minutes (15 min at 175 rpm and 15 minutes at 100 rpm). Then, single-cells prepared for FACS as in protocol A (Protocol B).

### FACS Sorting of eGFP+ and eGFP− Cells

Cells were sorted on a BD FACS Aria (DIVA version 5 software) cell sorter (BD Biosciences). Dead cells were stained with Propidium Iodide (PI) or Sytox Red (Invirogen, UK). They were detected and excluded from further analysis using either a 488 nm laser (617/25 bandpass filter) for PI, or a 633 nm laser (660/20 bandpass filter) for Sytox Red. Debris were also excluded from further analysis based on forward scatter versus side scatter plot. The viable single-cell population was analyzed on the basis of eGFP fluorescence intensity. A 488 nm laser was used for excitation and fluorescent signal was detected at a 530/30 nm bandpass. A control sample (isolated duodenal single cells from a CD-1/wt mouse) was used to establish the background level of autofluorescence and permit gating of the most intense fluorescent cells. Finally, living cells were sorted (directly into lysis buffer) into two populations, eGFP+ cells and equal number of eGFP− cells.

### RNA Isolation/RNA (cDNA) Amplification

RNA was prepared from sorted cells using RNA aqueous micro kit (AM1931, Ambion, UK) according to manufacturer’s protocol. Briefly, eGFP+ and eGFP− cells were sorted directly into 500 µl of cell lysis buffer. Then, 250 µl of 100% ethanol were added to the lysate and the mixture was passed through a micro filter cartridge. RNA bound to the filter was washed twice and eluted to a final volume of 20 µl. In order to confirm the integrity of RNA and calculate its relative concentration, we used RNA 6000 Pico Chip Assay on an Agilent 2100 Bioanalyzer (Agilent Technologies). RNA was treated with DNAseI (Ambion, UK) before reverse transcription to prevent contamination from genomic DNA.

For amplification of cDNA generated by RNA extracted from sorted cells, we used the Ovation Pico WTA System (Nugen), which requires as template 500 pg-50 ng of RNA. Amplification started with 5 µl of RNA as template (total amount of RNA was 2–5 ng). Amplified cDNA was purified using Qiagen PCR Purification Kit (Qiagen, UK), eluted at a final volume of 30 µl and nucleic acid concentration was measured using Nanodrop (Nanodrop Technologies).

### Semi-quantitative Reverse-transcription PCR (RT-PCR) Analysis

Reverse transcriptase PCR was performed using unamplified RNA from sorted cells. cDNA was synthesized from RNA using Superscript III reverse transcriptase (Invitrogen, UK). In parallel, identical reactions were setup in which Superscript enzyme was omitted and replaced by ddH_2_O. These samples were used as no reverse transcriptase (–RT) controls, to ensure that PCR products were not a result of genomic DNA contamination. The final volume of RT+ and RT- reactions were 20 µl of cDNA and 1 µl was used as template in each PCR reaction except for PCR for CB1 in which 1.5 µl was used as starting material. Amplified cDNA was diluted 1/50 and 1 µl was used as template in each reaction, except RT-PCR for Gpr119 (1.2 µl), Gpr120 (1.2 µl), CB1 (1.2 µl) and Gpr43 (1.2 µl). RNA that had not been amplified was diluted accordingly and served as negative (-RT) control. The quantity of starting cDNA template from eGFP+ and eGFP− samples was normalized using 18S rRNA housekeeping gene. PCR was performed using Biotaq (Bioline) and a typical mastermix for each reaction contained 10x NH_4_ reaction buffer (670 mM Tris-HCl pH 8.8, 160 mM (NH_4_)_2_SO_4_, 100 mM KCl, 0.1% stabilizer), 3 mM MgCl_2_, 0.8 mM dNTPs, 4% DMSO, 0.8 µΜ of each primer,1.75 Units of Biotaq and H_2_0 up to 25 µl. Oligomers used in the study are listed in Table S1. Reaction details (annealing temperature, cycles) are listed in Table S2. RT-PCR products were analyzed by electrophoresis on a 2% TBE (Tris/Borate/EDTA buffer) agarose gel. Sequencing of PCR amplicons was performed at University of Manchester Sequencing Core Facility.

## Supporting Information

Figure S1 **Imaging of eGFP+ and eGFP− cells pre- and post- FACS.** A: Imaging of dissociated duodenal single-cells. eGFP+ were less than 1% of total cell population and showed different levels of eGFP fluorescence. B: Imaging of sorted eGFP+ cells. Sorted eGFP+ cells showing eGFP fluorescence. C: Imaging of sorted eGFP− cells. Fluorescent cells were absent. Imaging confirms the successful FACS sorting of a highly enriched population of eGFP+ cells (A, B, C).
(TIF)

Figure S2 **eGFP+ cells are enriched in I-cells whereas eGFP**− **cells are I-cells depleted.** A: Semi-quantitative RT-PCR analysis of Cck mRNA transcript in dissociated duodenal cells and eGFP− cells showed that the assay is sensitive enough to detect Cck mRNA transcript in the starting population where the I-cells represent <1% of the cell population. After 33 cycles of PCR Cck mRNA transcript was detected in the dissociated duodenal cells (INPUT), but was not present in eGFP− cells. 18S rRNA was used as loading control (21 cycles). B: Semi-quantitative analysis of Cck mRNA transcript levels revealed that eGFP+ cells are enriched in I-cells in comparison with the starting dissociated cell population (INPUT). Cck PCR products were amplified for 27 cycles to ensure that we avoid the plateau phase of the reaction for eGFP+ sample. The amount of starting templates are equal with these shown at Panel A. 18S rRNA was used as loading control (18 cycles). L  =  Hyperladder V (50–250 bp, Bioline, UK). Asterisk indicates primer dimers.
(TIF)

Table S1 **Oligomers used in the study.** Primer sequences used for RT-PCR and the expected size (bp) of the amplicons are presented. For each GPCR, two sets of primers were used. Primer sequences for GPCRs RT-PCR analysis from amplified cDNA (Figure 5) obtained from PrimerBank (Harvard,USA) [50].
(DOC)

Table S2 **PCR cycling parameters.** Target names of primer pairs are listed along with the figure number in brackets where corresponding results are shown. The initial denaturation variables and final extension variables for all reactions were 95°C for 5 mins and 72°C for 10 min, respectively. Data for CB1 shown in figure 5 was generated using a split cycling program where cycles 1–12 are shown in row CB1 A and cycles >12 are shown in CB1 row B.
(DOC)
