# Supplementary figures and images for: Duodenal Enteroendocrine I-Cells Contain mRNA Transcripts Encoding Key Endocannabinoid and Fatty Acid Receptors
Source: PLoS One. 2012 Aug 2;7(8):e42373. doi: 10.1371/journal.pone.0042373 (PMC3410929; doi:10.1371/journal.pone.0042373)

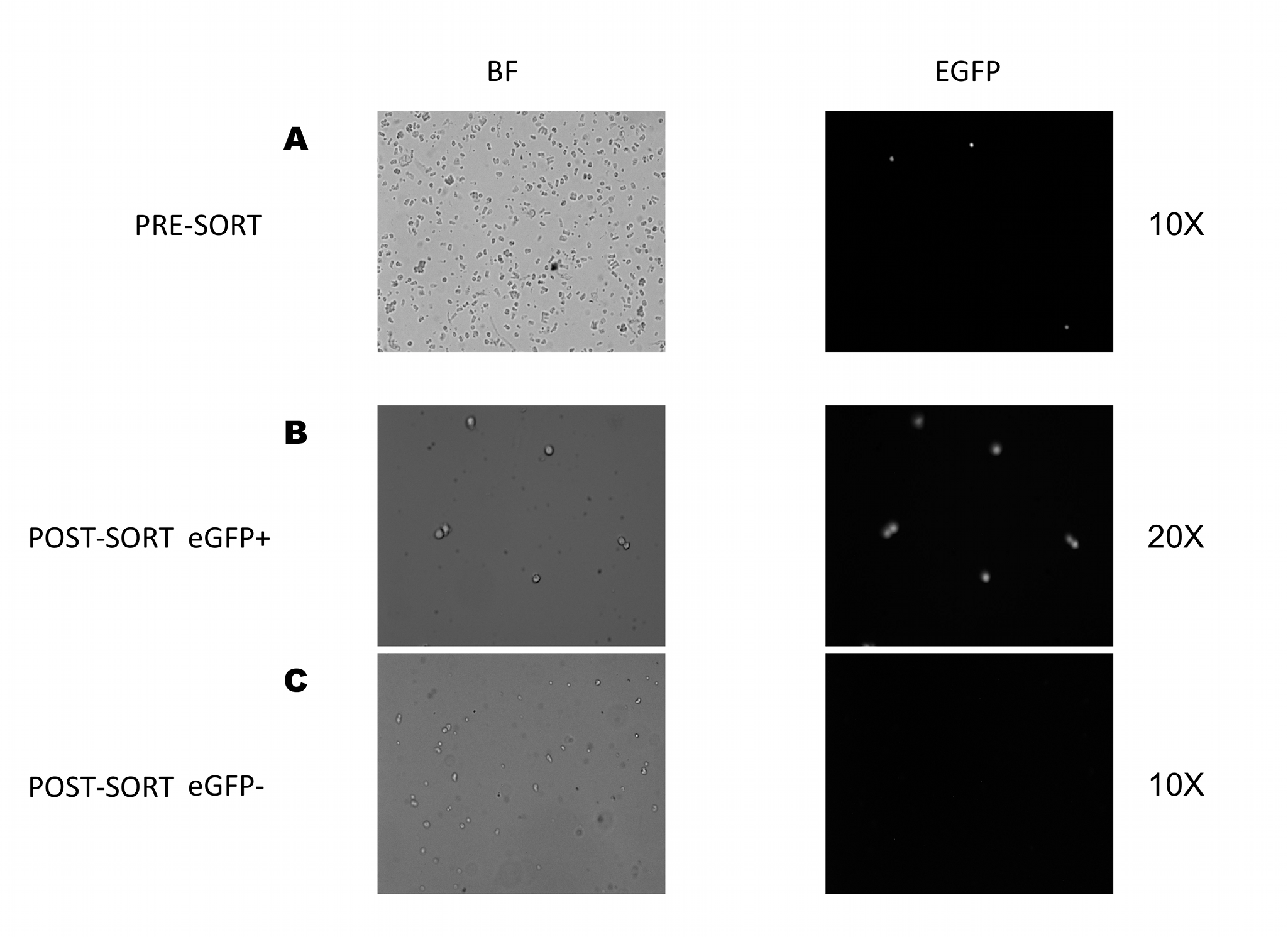

Supplement: Figure S1 — Imaging of eGFP+ and eGFP− cells pre- and post- FACS. A: Imaging of dissociated duodenal single-cells. eGFP+ were less than 1% of total cell population and showed different levels of eGFP fluorescence. B: Imaging of sorted eGFP+ cells. Sorted eGFP+ cells showing eGFP fluorescence. C: Imaging of sorted eGFP− cells. Fluorescent cells were absent. Imaging confirms the successful FACS sorting of a highly enriched population of eGFP+ cells (A, B, C). (TIF) [file pone.0042373.s001.tif]

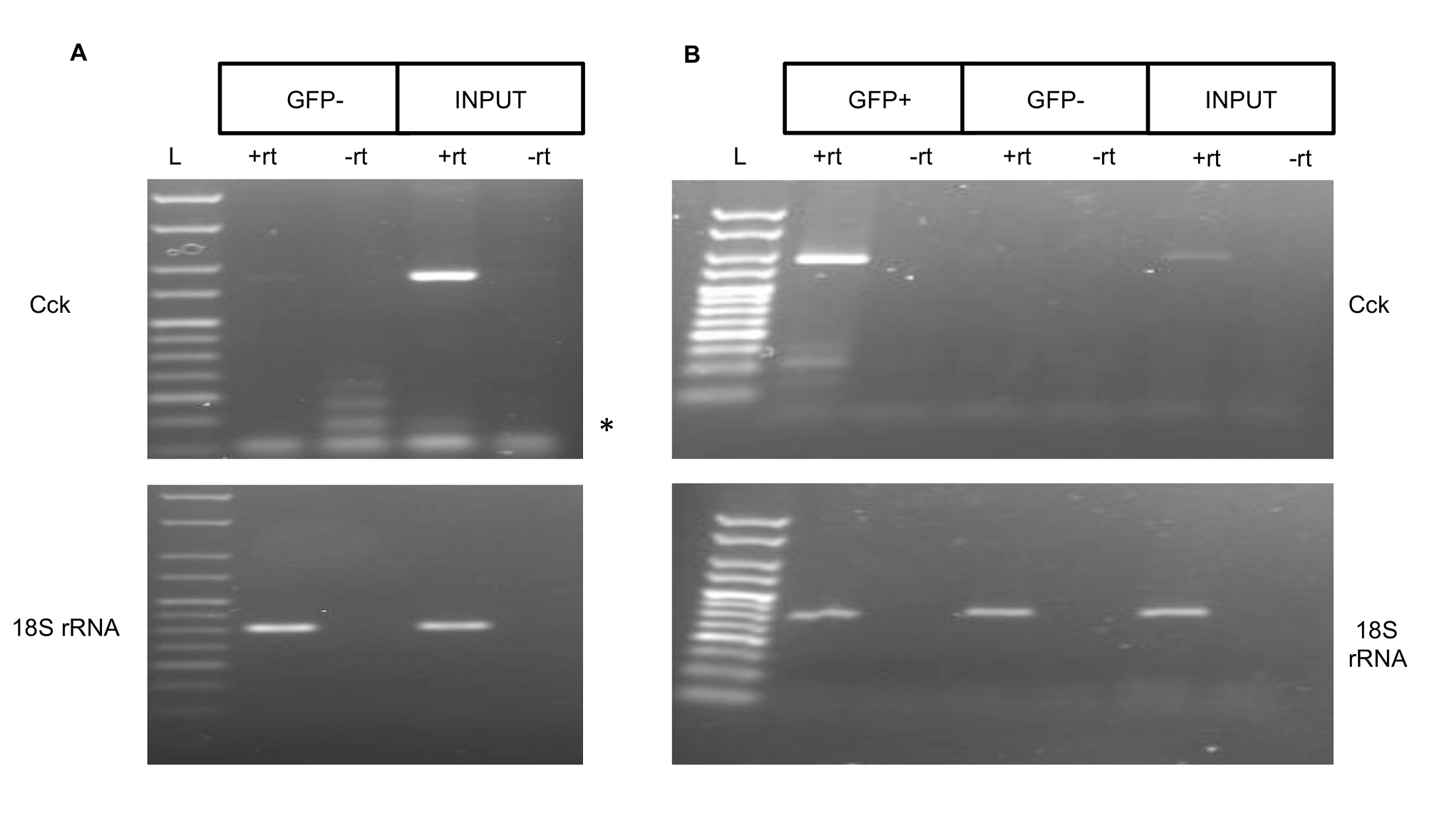

Supplement: Figure S2 — eGFP+ cells are enriched in I-cells whereas eGFP− cells are I-cells depleted. A: Semi-quantitative RT-PCR analysis of Cck mRNA transcript in dissociated duodenal cells and eGFP− cells showed that the assay is sensitive enough to detect Cck mRNA transcript in the starting population where the I-cells represent <1% of the cell population. After 33 cycles of PCR Cck mRNA transcript was detected in the dissociated duodenal cells (INPUT), but was not present in eGFP− cells. 18S rRNA was used as loading control (21 cycles). B: Semi-quantitative analysis of Cck mRNA transcript levels revealed that eGFP+ cells are enriched in I-cells in comparison with the starting dissociated cell population (INPUT). Cck PCR products were amplified for 27 cycles to ensure that we avoid the plateau phase of the reaction for eGFP+ sample. The amount of starting templates are equal with these shown at Panel A. 18S rRNA was used as loading control (18 cycles). L = Hyperladder V (50–250 bp, Bioline, UK). Asterisk indicates primer dimers. (TIF) [file pone.0042373.s002.tif]
