# Supplementary material for: Duodenal Enteroendocrine I-Cells Contain mRNA Transcripts Encoding Key Endocannabinoid and Fatty Acid Receptors
Source: PLoS One. 2012 Aug 2;7(8):e42373. doi: 10.1371/journal.pone.0042373 (PMC3410929; doi:10.1371/journal.pone.0042373)
Supplement: Table S1 — Oligomers used in the study. Primer sequences used for RT-PCR and the expected size (bp) of the amplicons are presented. For each GPCR, two sets of primers were used. Primer sequences for GPCRs RT-PCR analysis from amplified cDNA (Figure 5) obtained from PrimerBank (Harvard,USA) [50]. (DOC) [file pone.0042373.s003.doc]

| **Target** | **Forward Primer (5🡪3)** | **Reverse Primer (5🡪3)** | **Amplicon Size (bp)** |
| --- | --- | --- | --- |
| **18S rRNA** | GTAACCCGTTGAACCCCATT | CCATCCAATCGGTAGTAGCG | 151 |
| ***Cck*** | CGCTGGAACTCGCCAAGCCA | GCGGCCAGAAGGAGCTTTGC | 270 |
| ***Akp3*** | CTCATCTCCAACATGGAC | TGCTTAGCACTTTCACGG | 334 |
| ***Muc2*** | ACGATGCCTACACCAAGGTC | TGATCTTCTGCATGTTCCCA | 210 |
| **eGFP** | ACCCTCGTGACCACCCTGACCTAC | CGTCCTTGAAGAAGATGGTGCG | 133 |
| ***Gpr40/Ffar1***  **(Fig4)** | CAGTGTCCCACGCTAAACT | GGCAGAAAGAAGAGCAGAAT | 493 |
| ***Gpr40/Ffar1***  **(Fig5)** | ATCCGAGGCGCAGTGTCCCA | AGCCTCCGCCTGCGTAGAGG | 208 |
| ***Gpr41/Ffar3***  **(Fig4)** | AGTGCCAGTTGTCCAATACTC | GAAGAGGGAGGTGAGGTAAAT | 337 |
| ***Gpr41/Ffar3***  **(Fig5)** | TTGCTAAACCTGACCATTTCGG | GATAGGCCACGCTCAGAAAAC | 199 |
| ***Gpr43/Ffar2*** | CTTGATCCTCACGGCCTACAT | CCAGGGTCAGATTAAGCAGGAG | 137 |
| ***Gpr119***  **(Fig4)** | GCTGATTGCCTTTGACAGAT | GAGTGGGTTGAGTAGGGAGTT | 541 |
| ***Gpr119***  **(Fig5)** | TGGCAGAGGGAGGTTCGGCA | TCCTGCAGCGTCTTAGCCATCG | 177 |
| ***Gpr120/O3far1***  **(Fig4)** | TCTGCCACCTGCTCTTCTA | TTGTTGGGACACTCGGAT | 446 |
| ***Gpr120/O3far1***  **(Fig5)** | ACCAAGTCAATCGCACCCAC | GTGAGACGACAAAGATGAGCC | 111 |
| ***CB1***  **(Fig4)** | CGGGGGATGCGAAGGGGTTC | ACCGTGAAGGTGCCCAGGGT | 472 |
| ***CB1***  **(Fig5)** | CGGGGGATGCGAAGGGGTTC | GCCCACGTAGAGGAGGTCTGTGG | 149 |
