# Supplementary material for: Duodenal Enteroendocrine I-Cells Contain mRNA Transcripts Encoding Key Endocannabinoid and Fatty Acid Receptors
Source: PLoS One. 2012 Aug 2;7(8):e42373. doi: 10.1371/journal.pone.0042373 (PMC3410929; doi:10.1371/journal.pone.0042373)
Supplement: Table S2 — PCR cycling parameters. Target names of primer pairs are listed along with the figure number in brackets where corresponding results are shown. The initial denaturation variables and final extension variables for all reactions were 95°C for 5 mins and 72°C for 10 min, respectively. Data for CB1 shown in figure 5 was generated using a split cycling program where cycles 1–12 are shown in row CB1 A and cycles >12 are shown in CB1 row B. (DOC) [file pone.0042373.s004.doc]

| **Primer pair**  **(figure)** | | **Denaturation** | **Annealing** | **Extension** | **Number of Cycles** |
| --- | --- | --- | --- | --- | --- |
| ***Gpr40/Ffar1***  **(Fig 4)** | | 94 °C 60sec | 54°C 60sec | 72°C 60sec | 41 |
| ***Gpr40/Ffar1***  **(Fig 5)** | | 94°C 30sec | 62°C 30sec | 72°C 30sec | Low 29  High32 |
| ***Gpr41/Ffar3***  **(Fig4)** | | 94°C 60sec | 55°C 60sec | 72°C 60sec | 41 |
| ***Gpr41/Ffar3***  **(Fig5)** | | 94°C 30sec | 60°C 30sec | 72°C 30sec | Low 30  High 34 |
| ***Gpr43/Ffar2***  **(Fig4)** | | 94°C 30sec | 61°C 30sec | 72°C 30sec | 39 |
| ***Gpr43/Ffar2***  **(Fig5)** | | 94°C 30sec | 61°C 30sec | 72°C 30sec | Low 31  High 35 |
| ***Gpr119***  **(Fig4)** | | 94°C 60sec | 56.5°C 60sec | 72°C 60sec | 41 |
| ***Gpr119***  **(Fig5)** | | 94°C 30sec | 61.5°C 30sec | 72°C 30sec | Low 31  High 35 |
| ***Gpr120/O3far1*** **(Fig4)** | | 94°C 60sec | 55.5°C 60sec | 72°C 60sec | 42 |
| ***Gpr120/O3far1*** **(Fig5)** | | 94°C 30sec | 62°C 30sec | 72°C 30sec | Low 31  High 35 |
| ***CB1***  **(Fig4)** | | 94°C 60sec | 60.5°C 60sec | 72°C 60sec | 43 |
| ***CB1***  **(Fig5)** | A | 94°C 30sec | 68°C 30sec | 72°C 30sec | Low 32  High 36 |
|  | B | 94°C 30sec | 64°C 30sec | 72°C 30sec |  |
| ***Cck***  **(Fig4)** | | 94°C 45sec | 60°C 45sec | 72°C 45sec | 30 |
| ***Cck***  **(Fig5)** | | 94°C 45sec | 60°C 45sec | 72°C 45sec | Low 21  High 24 |
| ***Cck***  **(Fig S2)** | | 94°C 45sec | 60°C 45sec | 72°C 45sec | Fig S2A 33  Fig S2B 27 |
| ***eGFP***  **(Fig 5)** | | 94°C 30sec | 56°C 30sec | 72°C 30sec | Low 21  High 24 |
| ***Akp3***  **(Fig4)** | | 94°C 45sec | 53.5°C 45sec | 72°C 45sec | 35 |
| ***Akp3***  **(Fig5)** | | 94°C 45sec | 53.5°C 45sec | 72°C 45sec | Low 22  High 25 |
| ***Muc2***  **(Fig5)** | | 94°C 30sec | 56°C 30sec | 72°C 30sec | Low 23  High 26 |
| ***18S rRNA***  **(Fig4)** | | 94°C 30sec | 58°C 30sec | 72°C 30sec | 24 |
| ***18S rRNA***  **(Fig5)** | | 94°C 30sec | 58°C 30sec | 72°C 30sec | Low 14  High 18 |
| ***18S rRNA***  **(Fig S2)** | | 94°C 30sec | 58°C 30sec | 72°C 30sec | Fig S2A 22  Fig S2B 20 |
